# Hospital-Based Surveillance of Respiratory Viruses Among Children Under Five Years of Age with ARI and SARI in Eastern UP, India

**DOI:** 10.3390/v17010027

**Published:** 2024-12-28

**Authors:** Hirawati Deval, Mitali Srivastava, Neha Srivastava, Niraj Kumar, Aman Agarwal, Varsha Potdar, Anita Mehta, Bhoopendra Sharma, Rohit Beniwal, Rajeev Singh, Amresh Kumar Singh, Vivek Gaur, Mahima Mittal, Gaurav Raj Dwivedi, Sthita Pragnya Behera, Asif Kavathekar, Sanjay Prajapati, Sachin Yadav, Dipti Gautam, Nalin Kumar, Asif Iqbal, Rajni Kant, Manoj Murhekar

**Affiliations:** 1 Molecular Biology Division, ICMR-Regional Medical Research Centre, Gorakhpur 273013, India; mitali.srivastava710@gmail.com (M.S.); niraj.kumar.cdri@gmail.com (N.K.); asif1gtpl@gmail.com (A.K.); sanjayprajapati49@gmail.com (S.P.); yadavsachin6352@gmail.com (S.Y.); diptigautam446@gmail.com (D.G.); nalingkp@gmail.com (N.K.); asifiqbal2010@rediffmail.com (A.I.); 2 ICMR-Regional Medical Research Centre, Gorakhpur 273013, India; nehasrivastava2992@gmail.com (N.S.); amanstatistician01@gmail.com (A.A.); rbeniwal1111@gmail.com (R.B.); rajeevbt22@gmail.com (R.S.); grdnikhil@gmail.com (G.R.D.); sp.behera1@gmail.com (S.P.B.); rajnikant.srivastava@gmail.com (R.K.); 3 National Influenza Centre, ICMR-National Institute of Virology, Pune 411001, India; potdarvarsha9@gmail.com; 4 Department of Paediatrics, BRD Medical College, Gorakhpur 273013, India; 25anitamehta@gmail.com (A.M.); drbhoopy@rediffmail.com (B.S.); 5 Department of Microbiology, BRD Medical College, Gorakhpur 273013, India; amresh.sgpgi@gmail.com (A.K.S.); vivekbrdmc@gmail.com (V.G.); 6 Department of Paediatrics, AIIMS, Gorakhpur 273008, India; 7 ICMR-National Institute of Epidemiology, Chennai 600077, India; mmurhekar@gmail.com

**Keywords:** ARI, human metapneumovirus (hMPV), parainfluenza virus (PIV), respiratory syncytial virus (RSV), severe acute respiratory infection, under 5 children

## Abstract

Acute respiratory infections (ARIs) are a leading cause of death in children under five globally. The seasonal trends and profiles of respiratory viruses vary by region and season. Due to limited information and the population’s vulnerability, we conducted the hospital-based surveillance of respiratory viruses in Eastern Uttar Pradesh. Throat and nasal swabs were collected from outpatients and inpatients in the Department of Paediatrics, Baba Raghav Das (BRD) Medical College, Gorakhpur, between May 2022 and April 2023. A total of 943 samples from children aged 1 to 60 months were tested using multiplex real-time PCR for respiratory viruses in cases of ARI and SARI. Out of 943 samples tested, the highest positivity was found for parainfluenza virus [105 (11.13%) PIV-1 (79), PIV-2 (18), PIV-4 (18)], followed by adenovirus [82 (8.7%), RSV-B, [68 (7.21%)], influenza-A [46(4.9%): H1N1 = 29, H3N2 = 14), SARS CoV-2 [28 (3%)], hMPV [13(1.4%), RSV-A [4 (0.42%), and influenza-B (Victoria lineage) 1 (0.10%). The maximum positivity of respiratory viruses was seen in children between 1 to 12 months. The wide variation in prevalence of these respiratory viruses was seen in different seasons. This study enhances understanding of the seasonal and clinical trends of respiratory virus circulation and co-infections in Eastern Uttar Pradesh. The findings highlight the importance of targeted interventions to reduce the burden of respiratory infections in this region.

## 1. Introduction

Acute respiratory infection (ARI) poses a significant challenge to public health, because of its diverse etiology impacting the human airways [1]. While individuals of all age groups can be affected, young children are particularly more vulnerable [2]. Among infants, viral agents are primary contributors to ARI and pneumonia. Understanding the roles of these viral pathogens is complex, given the wide range of viruses associated with severe childhood ARI, including influenza virus A (Flu-A), influenza virus B (Flu-B), respiratory syncytial virus (RSV), parainfluenza virus (PIV), human metapneumovirus (hMPV), human rhinovirus (HRV), and human adenovirus (HAdV) [3,4,5,6,7].

The prevalence and circulation of these respiratory viruses vary considerably with seasonality, geographical region, and occupation. Ascertaining the viral pathogens causing ARI and severe ARI (SARI) is particularly challenging in resource-limited settings.

India, with its vast population, diverse geography, and seasonal variations, bears one of the highest global burdens of pneumonia and childhood mortality due to ARI [8]. As per the national family health survey (NFHS-5) conducted during 2019–2021, about 14.3% of deaths in young children and 15.9% of deaths in children aged between 1 to 5 were due to ARI. Among all states in India, Uttar Pradesh (UP) is a major contributor to the annual national burden of ARI cases among children under five years of age (NFHS-5) [9].

Because of several risk factors, Eastern UP favors the transmission of viral respiratory pathogens. It also shares an international border with Nepal and a national border with Bihar, which increases the risk of cross-border transmission of ARI and SARI pathogens [10]. Additionally, it is a flood-prone area subject to water logging, along with having a warm, humid environment and engaging in practices like poultry and livestock farming, which enhance the risk [11,12]. Understanding the etiological agents of ARI and SARI in this region is essential for surveillance, diagnostics, and treatment, and to explore the relationships between ARI in children and local demographic, socioeconomic, and environmental factors. This knowledge is crucial for shaping national public health policies on ARI and SARI.

While many viral infections are common among children with ARIs, recent advancements in molecular biology techniques, such as multiplex PCR, have enabled the simultaneous detection of multiple viruses with high sensitivity and specificity [5,13,14]. This heightened sensitivity has led to the detection of viruses even in asymptomatic cases [15,16]. Investigating severe acute respiratory infections (SARIs) in children requires comprehensive research into the risk factors for lower respiratory tract infections (LRTIs). Given the susceptibility of children to ARI, identifying the specific viruses accountable for these infections is essential. Furthermore, it is important to determine whether respiratory viruses act alone or in conjunction with co-infections to cause severe ARI.

To address these challenges, we conducted a hospital-based surveillance study to investigate respiratory viruses among children under 5. Our goal was to determine the frequency and consequences of both single and multiple respiratory virus infections among both outpatients and hospitalized children with acute respiratory infections (ARIs).

## 2. Methods

### 2.1. Study Design: Cross-Sectional Study

Study site and study population

The study included children under five years of age with symptoms of acute respiratory illness (ARI) and severe acute respiratory illness (SARI) who attended the outpatient department (OPD) or were admitted to the inpatient department (IPD) of the Department of Paediatrics, Baba Raghav Das (BRD) Medical College, Gorakhpur, UP, India.

Nasal and throat swabs (NS/TSs) were collected by trained staff weekly for one year (May 2022–April 2023). Every week, 20 ARI and SARI samples were collected based on cases (approx. 1000 samples per year). Samples were transported to ICMR-RMRC Gorakhpur, maintaining the cold chain for laboratory investigations. The biochemical and clinical data of the patients were also collected from the hospital records.

### 2.2. Ethics Approval

The study protocol was approved by the institutional ethics committee of ICMR-Regional Medical Research Center (ICMR-RMRCGKP), Gorakhpur (EC ID: RMRCGKP/EC/2020/1.1). Given that the participants were children aged under 5 years, their parents or guardians were informed about the study in their local language and provided with a patient information sheet (in their local language). Written assent was obtained from the parents or guardians of the children before enrolling them in the study, ensuring compliance with ethical standards and the protection of participants’ rights. All identifiers were decoded for analysis. The samples were collected by trained medical professionals.

### 2.3. Case Definition of ARI

Acute respiratory infection (ARI) was defined for participant enrolment as an illness presenting in the OPD with the acute onset (within 7 days) of any two of the following symptoms: fever, cough, sore throat, nasal congestion, or shortness of breath. The World Health Organization (WHO) case definition was employed to characterize severe acute respiratory infections (SARIs), which encompassed cases featuring a recent history of fever (or measured fever of ≥38 °C) and cough that began within the last week and necessitated overnight hospitalization. To define SARI in children, a doctor’s diagnosis of an acute lower respiratory infection (such as pneumonia, bronchitis, bronchiolitis, or sepsis) with overnight hospitalization was utilized [17,18,19].

### 2.4. Hospital Case Enrollment and Data Collection

From the hospital, children under 5 years of age presenting symptoms of ARI were recruited from the Outpatient Department (OPD), while SARI patients were enrolled from the Paediatric Intensive Care Unit (PICU), HDU (High Dependency Unit), and Emergency Ward. Patients who met the case criteria and whose parents or guardians provided permission were included in the research study. A standardized case report form was utilized to collect information of each patient’s clinical and epidemiological details.

### 2.5. Specimen Collection

Clinical sample collection, transportation, and storage were undertaken in accordance with WHO recommendations [20]. Nasal and/or throat respiratory specimens were collected by the trained technicians from enrolled ARI and SARI cases using a viral transport medium (GeneBio Healthcare Pvt Ltd., Lucknow, India). These specimens were then transported to the ICMR-RMRC, Gorakhpur, Molecular Biology laboratory while maintaining the cold chain. Samples that were not immediately examined were stored at −80 °C to preserve their integrity.

### 2.6. Molecular Detection

Nucleic acid extraction was performed using the QIAamp Viral RNA Mini kit (Qiagen, Hilden, Germany). The extracted nucleic acids were then subjected to detection using a panel of respiratory viruses, including Flu-A (H1N1, H3N2), Flu-B (Yamagata, Victoria lineages), SARS-CoV-2, respiratory syncytial virus (RSV-A, RSV-B), adenovirus (HAdV), parainfluenza virus (PIV 1–4), human metapneumovirus (hMPV), and human rhinovirus (HRV). Detection was achieved through real-time reverse transcriptase PCR (rRT-PCR) targeting specific genes, including the house-keeping gene RNase P. The protocols followed for rRT-PCR were in accordance with the guidelines provided by the WHO [20] and CDC [21]. Detailed lists of primers and probes used in the multiplex PCR are mentioned in Supplementary Table S1. For simultaneous detection of Flu-A, Flu-B, and SARS-CoV-2, samples were tested by a two-step rRT–PCR kit designed by the National Influenza Centre (NIC), NIV, Pune, India [18].

Nucleic acid amplification was conducted using a one-step RT-PCR kit (qRT-PCR SuperScript III kit, Invitrogen, Seattle, Washington, DC, USA). The reaction mixture consisted of 10 µM of each forward and reverse primer, 5 µM of TaqMan probe, 12.5 µL of 2X buffer, 0.5 µL of SuperScript III enzyme, and 5 µL of nucleic acid templates, resulting in a total volume of 25 µL for each PCR reaction. Thermal cycling was performed with the following conditions: reverse transcription at 50 °C for 30 min, initial denaturation at 95 °C for 5 min, followed by 45 cycles of two stages (15 s at 95 °C, and 30 s at 55 °C, with data acquisition).

### 2.7. Statistical Analysis

Patient data were entered into Epi-info 7.2.5.0 at the hospital. Continuous variables are reported as mean and standard deviation, while categorical variables are represented as numbers with percentages. Bivariate logistic regression analysis was performed to establish the association between (a) dichotomous categorical variables and (b) categorical and continuous variables. Multivariable logistic regression analysis was also performed to control for the effect of variables found to be significant in bivariate logistic regression analysis. Results of logistic regression analysis (bivariate and multivariable) are represented as odds ratio and adjusted odds ratio, respectively, along with the 95% confidence limit and significance value. The cut-off for statistical significance was <0.05 throughout the statistical analysis. Stata ver. 13.0 was used for statistical analysis. Graphs were made using MS Office 2010.

## 3. Results

### 3.1. Patient Characteristics:

During the period spanning from May 2022 to April 2023, samples from 943 ARI and SARI patients were collected. Among these 943 patients, 620 (65.75%) were male and 323 (34.25%) were female. The age distributions of the patients ranged from 0 to 5 years, and the majority (56.52%) were between 0 and 1 year old. The socio-demographic and clinical characteristics of recruited ARI (n = 611) and SARI (n = 332) patients are presented in Table 1.

### 3.2. Viral Etiologies in ARI and SARI Cases

All 943 samples underwent screening for the presence of respiratory viruses using real-time PCR (Supplementary Table S1). Results revealed the highest positivity for parainfluenza virus [105 (11.13%): PIV-1 (79), PIV-2 (18), PIV-4 (18)], followed by HAdV [82 (8.7%)], RSV-B [68 (7.2%)], Flu-A [46 (4.9%): (H1N1 = 29, H3N2 = 14)], SARS-CoV-2 [28 (3%)], hMPV [13 (1.4%)], HRV [10 (1.06%)], and RSV-A [4 (0.4%)], and 1 (0.1%) was positive for Flu-B (Victoria lineage) (Table 2).

Among ARI cases, the highest positivity was found for PIV [84 (13.7%): PIV-1 (65), PIV-2 (14), PIV-4 (5)], followed by HAdV [56 (9.17%)], Flu-A [36 (5.89%)], RSV-B [26 (4.26%)], SARS CoV-2 [23 (3.76%)], hMPV [12 (1.96%)], HRV [4 (0.42%)], and RSV-A [2 (0.33%)], and 1 (0.1%) was positive for Flu-B (Victoria lineage). Among SARI cases, the highest positivity was found for RSV-B [42 (12.65%)], followed by HAdV [26 (7.83%)], parainfluenza virus [21 (6.32%): PIV-1 (14), PIV-2 (4), PIV-4 (3)], Flu-A [10 (3.01%)], SARS-CoV-2 [5 (1.51%)], HRV [6 (0.64%)], RSV-A [2 (0.60%)], and hMPV [1 (0.30%)] (Table 2).

### 3.3. Comparative Analysis of Clinical Characteristics of ARI and SARI Cases Tested Positive for Viral Etiologies Versus Negative for Viral Etiologies

The analysis comparing the characteristics of patients with ARI and SARI who tested positive for viral etiologies versus those who tested negative are represented in Table 3 and Table 4. In ARI cases, a significant association was identified between viral positivity and exposure to farm animals. The unadjusted odds ratio (O.R.) was 4.3 (95% C.I.: 2.1–8.5, *p* value < 0.01), indicating that individuals exposed to farm animals had a substantially higher likelihood of testing positive for viral etiologies compared to those without such exposure. This association remained significant even after adjusting for confounding factors, with an adjusted odds ratio (A.O.R) of 3.4 (95% C.I.: 1.6–7.1, *p* value < 0.01). Additionally, a significant association was observed between having a similar illness in the family or neighborhood and viral positivity, with an OR of 2.7 (95% CI: 1.1–6.4, *p* value= 0.03) among ARI cases (Supplementary Table S2).

Similar to ARI cases, exposure to farm animals was also significantly associated with viral positivity among SARI cases (AOR: 4.3; 95% CI: 1.3–14.0, *p* = 0.02). The SARI cases with smokers in the family were more likely to suffer from severe viral respiratory infections requiring hospitalization (AOR: 9.2; 95% CI: 2.4–35.7, *p* < 0.01) (Supplementary Table S3).

### 3.4. Clinical Characteristics of ARI and SARI Cases Compared for Different Respiratory Viral Pathogens

In ARI patients, SARS CoV-2-positive cases consistently presented with symptoms of fever, cough, and sore throat. Similarly, in cases positive for Flu-A, sore throat was universally present, while fever and cough were reported in 63.9% of cases. Among hMPV-positive cases, the predominant symptoms included fever (100%), cough (100%), and nasal discharge (75%), while no cases exhibited abdominal pain, seizures, diarrhea, wheezing, or apnea. RSV-B positive cases exhibited fever (100%), cough (100%), and sore throat (96.2%) as the most common symptoms, with nasal discharge reported in 57.7% of cases. Some patients also presented with breathlessness (26.9%), body ache (3.8%), and vomiting (3.8%). In cases positive for PIV-1, fever (100%), cough (98.5%), and sore throat (98.5%) were prevalent symptoms. However, nasal discharge (50.8%), breathlessness (29.2%), apnea (7.7%), and chills/rigors (3.1%) were observed in only a minority of cases. Symptoms such as vomiting, abdominal pain, seizures, diarrhea, wheezing, and crepitation were absent in all PIV-1 positive cases. We found that in PIV-2 positive cases, fever and cough were common in all the patients, while nasal discharge was found in 42.9% of the cases, breathlessness in 28.6% of the cases, nasal flaring in 57.1% of the cases, and apnea in 14.3% of the positive cases. In PIV-4 positive cases, it was also found that sore throat, fever, and cough were the common symptoms, while nasal discharge (40%), chills/rigors (20%), breathlessness (20%), nasal flaring (60%), and apnea (20%) were present in few cases. It was found that in HAdV-positive cases, cough and fever were the common symptoms, while nasal discharge was present in 46.4% of the cases, breathlessness in 33.9% of the cases, nasal flaring in 55.4% of the cases, and apnea in 10.7% of the HAdV-positive cases. In human HRV-positive patients, fever, cough, sore throat, and breathlessness were the common symptoms, apnea was found in 50% of the patients, and nasal flaring was found in 75% of the patients (Supplementary Table S4).

In SARI patients, SARS-CoV-2-positive cases consistently exhibited symptoms of fever, cough, breathlessness, and wheezing. Additionally, sore throat and prior history of respiratory infection were found in 80% of cases. All SARS-CoV-2 patients required admission to the Intensive Care Unit (ICU) and received oxygen therapy. Of these patients, 80% recovered and were discharged, while 20% succumbed to the illness. In cases positive for Flu-A, common symptoms included fever (100%), cough (90%), sore throat (80%), breathlessness (90%), wheezing (100%), and apnea (50%). Chest X-rays were performed on 90% of patients, revealing heterogeneous capacities in 80% of cases. All Flu-A-positive patients required ICU admission, and 60% were successfully treated and discharged. hMPV-positive cases presented with symptoms of fever, cough, breathlessness, abdominal pain, diarrhea, and wheezing. However, all hMPV-positive patients recovered and were discharged. In RSV-A-positive cases, fever, cough, breathlessness, and wheezing were commonly observed symptoms. Like hMPV-positive cases, all RSV-A-positive patients recovered and were discharged (Supplementary Table S5).

In RSV-B-positive cases, patients commonly exhibited symptoms such as fever (95.2%), cough (95.2%), sore throat (78.6%), breathlessness (97.6%), wheezing (83.3%), and apnea (45.2%). A significant portion of these patients, 95.2%, required oxygen supplementation, and 90.5% underwent chest X-rays, with 76.2% diagnosed with heterogeneous capacities. The majority of RSV-B-positive patients (78.6%) were admitted to the ICU, where 78.6% of them recovered and were subsequently discharged. In PIV-1 positive cases, 85.7% of patients presented with fever and cough, while sore throat was reported in 64.3% of cases and breathlessness in all positive cases. Nasal flaring was observed in 35.7% of cases. All PIV-1-positive patients required ICU admission and received appropriate treatment, resulting in a recovery rate of 78.6%. Patient’s positive for HAdV exhibited symptoms including fever (100%), cough (96.2%), sore throat (88.5%), breathlessness (96.2%), wheezing (84.6%), and apnea (38.5%). All adenovirus-positive patients were admitted to the ICU, with 96.25% requiring oxygen therapy. Half of the patients (50%) recovered and were discharged following treatment. Patients positive for human rhinovirus exhibited symptoms including fever, cough, sore throat and breathlessness (100%), wheezing and apnea (66.7%), and nasal discharge (16.7%). All HRV-positive patients were admitted to the ICU, with 100% requiring oxygen therapy. Patients who unfortunately expired accounted for 66.7%, and 33.3% of patients left against medical advice (LAMA) (Supplementary Table S5).

### 3.5. Outcome of Viral Respiratory Pathogens in SARI Cases

The average duration of hospitalization for SARI patients was 12 days (11.9 ± 12.0 days), with a minimum stay of 1 day and a maximum of 75 days. The overall mortality rate among SARI patients was 12.3%, accounting for 41 out of 332 cases. Maximum mortality was observed in patients infected with HAdV (24.3%), followed by RSV-B (12.1%), HRV (9.76%), PIV-1 (4.8%), and PIV-4, and 2.4% mortality was observed in patients infected with PIV-4, SARS CoV-2, and Flu-A. There was no mortality in patients with Flu-B, hMPV, or RSV-A.

### 3.6. Seasonal Distribution of Respiratory Viral Pathogens

**Figure 1:**
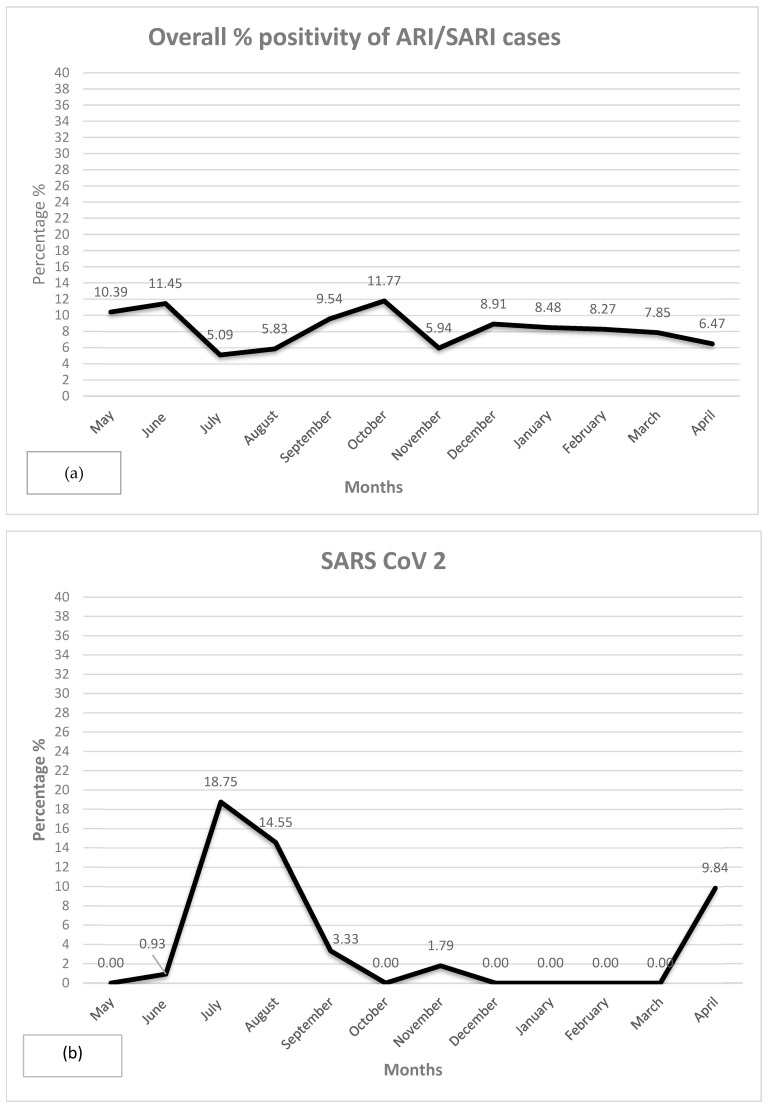

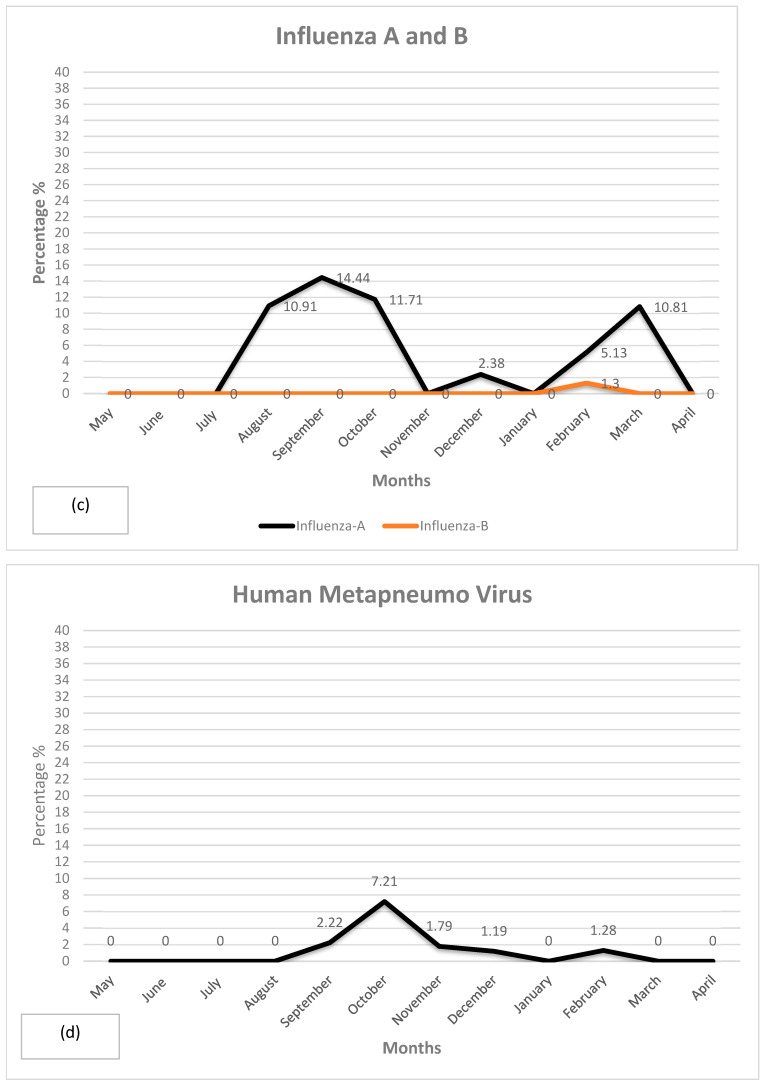

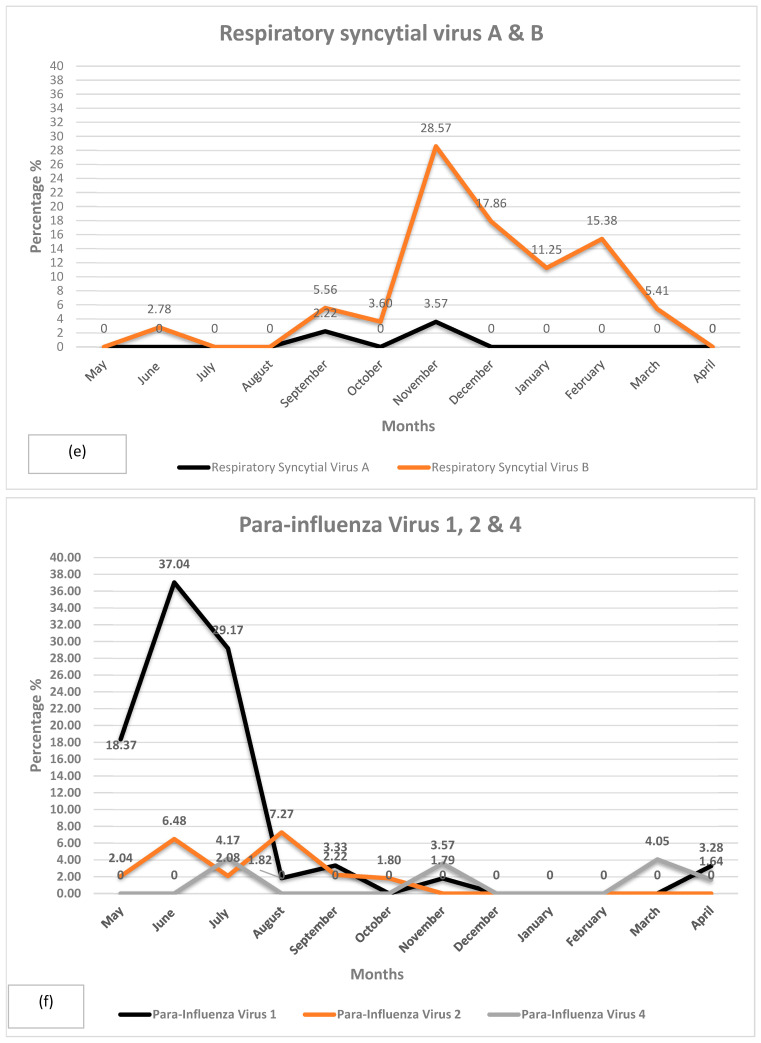

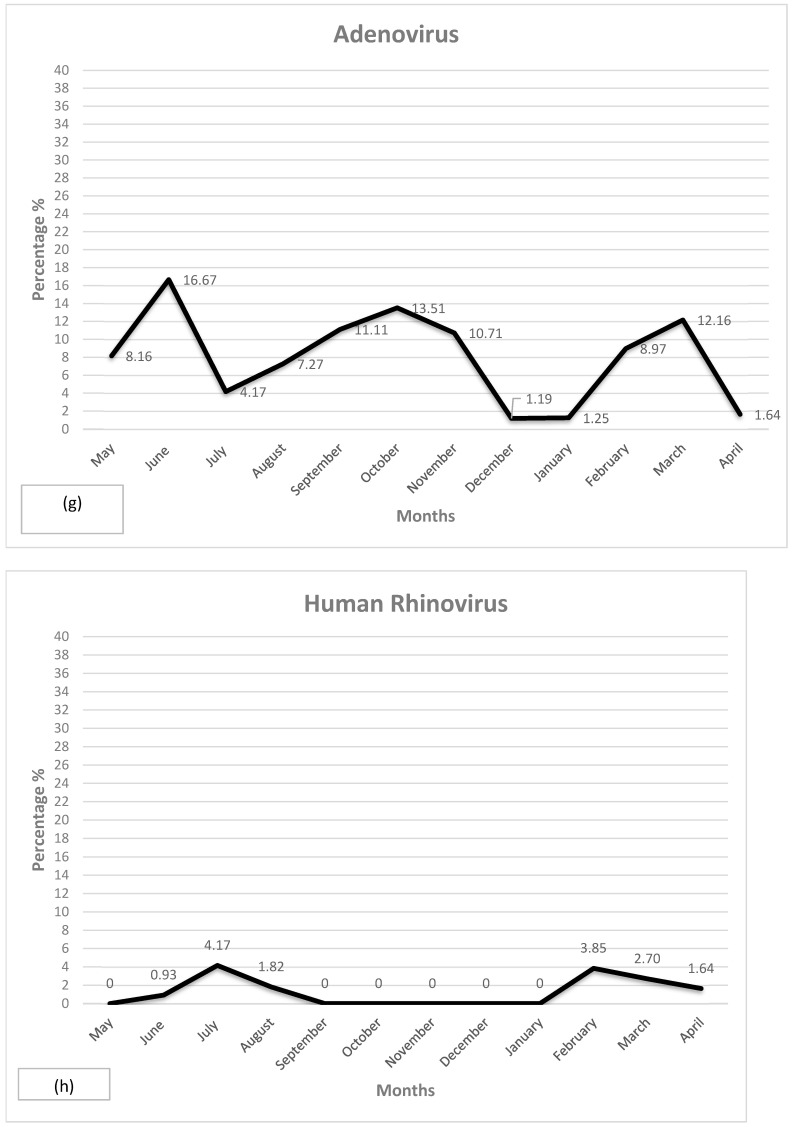
(**a**–**h**). Seasonal distribution of respiratory viral pathogens. (**a**) Overall % Positivity of ARI/SARI Cases–The positivity rate peaks in July (11.77%). (**b**) SARS-CoV-2–A high positivity rate is observed in July (18.75%) and August (14.55%), followed by a drop to 0% from October to March, with a small resurgence in April (9.84%). (**c**) Influenza A and B–Influenza A has two distinct peaks in October (14.44%) and April (10.81%), suggesting a bimodal seasonal pattern. Influenza B, with lower positivity, peaks in January (3.38%), showing less seasonal fluctuation. (**d**) Human Metapneumovirus–peaks in October (7.21%). The virus has minimal activity during most months, indicating a brief seasonal presence. (**e**) Respiratory Syncytial Virus (RSV) A & B–. RSV B shows a significant peak in October (28.57%), while RSV A has smaller peaks in September (5.56%) and March (4.10%), suggesting different seasonal patterns for each subtype. (**f**) Para-Influenza Virus 1, 2, & 4–Type 1 has a high peak in May (37.04%) with rapid decline afterward. Type 3 has a small peak in October (8.33%), while types 2 and 4 remain low throughout the months, indicating type-specific seasonality. (**g**) Adenovirus–Displays a fluctuating positivity rate for Adenovirus with peaks in August (16.67%) and January (11.11%), showing sporadic activity and scattered occurrences without a clear seasonal trend. (**h**) Human Rhinovirus–Shows the positivity rate of Human Rhinovirus, which remains low overall, with a small peak in October (4.17%) and slight increases in March (3.85%) and April (1.64%), indicating minor seasonal variations.

The overall positivity percentage of ARI/SARI cases peaks in July (11.45%), November (11.77%), and January (8.48%), with the lowest rate in June (0.09%) (Figure 1a). For SARS-CoV-2, the highest positivity rates occur in July (18.75%) and August (14.55%), with a resurgence in April (9.84%) following several months of low to no detection (Figure 1b). Flu-A and Flu-B show distinct patterns, with Flu-A peaking in August (14.44%) and December (11.71%), while Flu-B has a smaller peak in February (3.38%) (Figure 1c). hMPV reaches its peak in October (7.21%), with few to no cases detected in other months (Figure 1d). Significant activity is exhibited for RSV-B in October (28.57%), remaining relatively high through December (15.38%), while RSV-A shows smaller peaks in August (5.56%) and October (3.66%) (Figure 1e).

The parainfluenza viruses cause the highest burden of ARI/SARI, with PIV-1 peaking in July (37.04%) and August (29.17%), whereas PIV-2 and PIV-4 maintain much lower peaks (Figure 1f). HAdV peaks in July (16.67%) and December (13.51%), with additional lower peaks in June (8.16%) and April (12.16%) (Figure 1g). HRV peaks in July (4.17%) and March (3.85%), with no cases detected in several months, including May, October, November, December, January, and February (Figure 1h).

**Figure 2:**
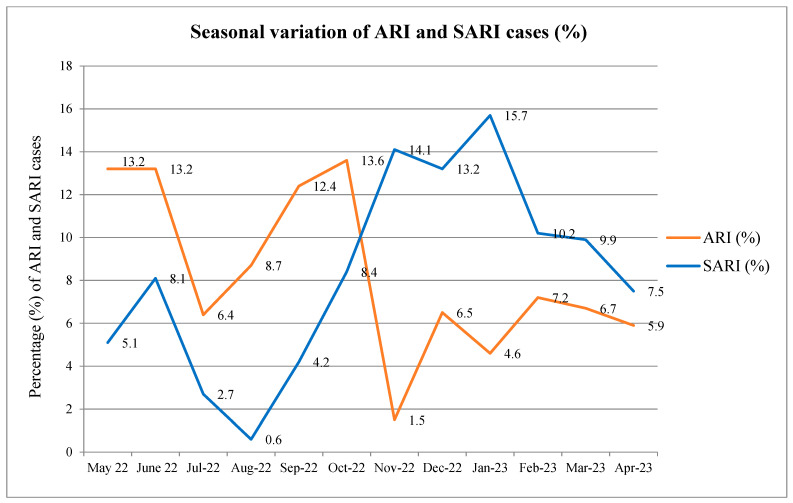
Seasonal distribution of ARI and SARI cases during May 2022–Apr 2023.

Figure 2 demonstrates the seasonal variation in ARI and SARI cases. During pre- and post-monsoon periods, the number of ARI cases reaches a peak, while the majority of SARI cases was observed during winter months.

### 3.7. Co-Infection of Respiratory Viruses in ARI/SARI Cases

**Figure 3:**
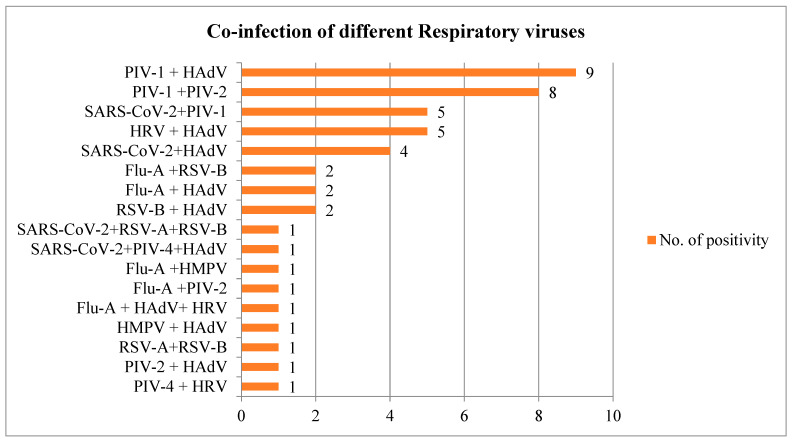
Co-infection of different respiratory viruses.

A total of 46 (14.9%) patients tested for more than one respiratory virus or had viral co-infection. Out of 46 co-infected cases, 34 were ARI and 12 were SARI. Among these cases, maximum co-infection was observed with HAdV and PIV i.e., 56.5% co-infection. Of this 56.5% PIV co-infection, the maximum was found between PIV-1 and HAdV, with 19.6%. PIV-1 also had additional co-infection cases with PIV-2, i.e., accounting for 17.4% of the total. Other notable co-infections included five cases (10.9%) of HRV combined with HAdV, and five cases (10.9%) of SARS-CoV-2 combined with PIV-1 (Figure 3).

Other co-infections found in the present study are mentioned in Figure 3. Among 46 cases with co-infections, no co-infections were observed with more than three viruses. Overall, these findings illustrate the diversity of respiratory virus co-infections observed alongside SARS-CoV-2.

Further, the patient’s characteristics and outcome were compared between mono-infected and co-infected cases (Table 3 and Table 4). The co-infected SARI patients were more likely to have seizures (OR (95%CI) 17.2 (1.4–207.1); *p*-value 0.02), requirement of continuous positive air pressure (CPAP) (OR (95%CI) 3.4 (0.9–12.3); *p*-value 0.05), and bronchodilators (OR (95%CI) 4.2 (0.7–25.6); *p*-value 0.13). Mortality was on the higher side in co-infected SARI patients in comparison to mono-infected SARI patients (OR (95%CI) 2.4 (0.6–9.3); *p*-value 0.20).

## 4. Discussion

India has a substantial burden of acute respiratory infections among all ages. The aim of the present study was to describe the frequency, clinical profile, and seasonal pattern of respiratory viruses in a hospital-based surveillance over a period of one year (2022–2023) in children under five years of age. The predominant causative agents in the present study were found to be PIV (11.13%) followed by adenovirus (8.7%) among all respiratory illness cases. The viral etiologies varied between ARI and SARI cases. PIV (13.74%) was the major viral etiology in ARI cases, followed by adenovirus (9.17%), while RSV-B (12.65%) and adenovirus (7.83%) contributed to the majority of severe ARI cases. In a study in Rajasthan [22], hMPV (25.7%) and influenza-A (19.9%) predominated in SARI cases, while in the present study hMPV and influenza-A accounted only for 1.4% and 4.9% of cases, respectively. In India, there are varied findings for the prevalence of viral etiologies in respiratory illness cases. A previously published comprehensive literature review observed RSV (26%) as a major etiology for respiratory illnesses among children above 5 years, followed by influenza (11%) and parainfluenza (10%) [23], while in South India RSV (45.69%) and rhinovirus (17.88%) were the most common viral etiologies for respiratory illnesses among children under 5 [24].

Understanding the clinical profiles presented by ARI and SARI cases is of paramount importance for clinicians to manage the mortality and morbidity in children. In our study, fever, cough, and cold were the most common symptoms among ARI and SARI cases, although ARI was most common among children aged 19–20 months, while SARI was most common among 9–10-month-old children. ARI most commonly presented with cough and nasal flaring, while SARI cases had breathlessness, abdominal pain, prior history of respiratory infection, and wheezing. Our study provides information on differential clinical presentations of ARI and SARI cases in different viral pathogen infections. The characteristics of the patients were compared with the viral etiology and it was found that in cases of both ARI and SARI, the patients who were exposed to farm animals were more likely to test positive for a viral etiology than those who were not exposed. Also, the SARI cases with smokers in the family were more likely to develop severe viral respiratory infections necessitating hospitalization.

The average hospitalization for SARI patients was 12 days (11.9 ± 12.0 days), with a minimum of 1 day and a maximum of 75 days. According to research, developing nations account for 99% of mortality in children under the age of five from lower respiratory tract infections caused by influenza [25]. Our study depicted that the overall mortality rate among patients was 12.3%. However, in a pan-India study [19], mortality of 4.7% was reported due to viral infections.

The climate of Eastern UP, India, is primarily humid subtropical with dry winters, and this may play a substantial role in different respiratory viruses’ transmission. During winter (November–February), RSV-B and HRV are common, followed by HAdV, while during the pre-monsoon period (March-May), SARS-CoV-2, Flu-A, and HAdV are the common viral etiologies among ARI/SARI cases. During monsoon summer (June-September), SARS-CoV-2, Flu-A, hMPV, PIV, and HAdV, followed by HRV, are the common etiologies. Flu-A, hMPV, and HAdV contribute significantly to ARI/SARI during the post-monsoon period (October). SARS-CoV-2 cases start in June, reach a peak in July (18.75%), and gradually decline in September. Flu-A cases start in August (10.91%), attain a peak in September (14.44%), and gradually decline after October (11.71%). hMPV cases start showing up in September, reach a peak in October, and then gradually decline with minimum cases in November-December. RSV-B attains a peak in November (28.57%) and gradually declines afterwards. PIV-1 starts presenting among ARI/SARI cases in May, attains a peak in June (37.04%), and gradually declines afterwards. HAdV is a common etiology through the year. HRV starts in February and peaks in July (4.2%), with no cases during September-January.

Our study is in line with a pan-India surveillance study by Potdar et al., from 2021 to 2022, who reported 4.1% to 12% positivity of influenza in <5-year SARI and ARI patients, respectively [19], while we found influenza positivity in 4.9% of patients, which is in the given range. Prior to the COVID-19 pandemic, seasonal patterns of respiratory virus circulation followed previously published reports, with annual peaks of influenza and RSV occurring in late autumn and winter months [26,27]. In our study also, RSV was more prevalent in winter months. RSV-B is very common among children aged less than 5 years, and we found 28.6% positivity in children, mainly in the late autumn and winter months. This is in concurrence with an earlier study [28], which also found RSV-B in the winter months and spring months, with 9.52% positivity. There was no positivity of PIV-3 in children with ARI and SARI cases in our study.

The findings of this study also support the positivity prevalence reported by another study [19], of 2.4% to 3.5% and 2% to 3.8% in SARI and ARI patients, respectively, if we matched the results with our age-group population. The rate of respiratory virus co-infections varied greatly between investigations worldwide [29,30,31]. A total of 46 (14.9%) co-infections were identified in the present study, which was lower than in a study of Rajasthan [22] and higher than in a pan-India study [19] in which they found 0.4% co-infections among SARI cases in children aged less than 5 years. The highest co-infection in our study was seen with HAdV and PIV (56.5%). Other co-infections were with SARS-CoV-2 (23.9%), followed by Flu-A and HRV (15.2%), RSV (13.0%), and hMPV (4.3%). When the characteristics of mono-infected and co-infected patients were compared, it was found that co-infected SARI patients were more likely to have seizures (O.R. (95% C.I.) 17.2 (1.4–207.1); *p*-value 0.02), require continuous positive air pressure (CPAP) (O.R. (95% C.I.) 3.4 (0.9–12.3); *p*-value 0.05), and use bronchodilators (OR (95% C.I.) 4.2 (0.7–25.6); *p*-value 0.13). Mortality in co-infected SARI patients was greater than in mono-infected SARI patients (O.R. (95% C.I.) 2.4 (0.6–9.3); *p*-value 0.20).

Young children, particularly infants, have immune systems that are still developing. In addition, their lungs and airways are smaller, making viruses that affect airways more of a threat. We observed that patients of age <2 years are most infected as compared to patients >2 years. According to another study [32], it was found that the respiratory viruses’ detection rate was highest in children aged up to one year. Another study [33] also indicated that maximum viral infections are observed in children less than five years of age.

Respiratory viruses, either alone or in combination with other viral infections, are a significant cause of morbidity in children. This study provides an insight into the frequency of detection of all 12 viruses by multiplex PCR from a total of 943 specimens, which was found to be 37.85% (357/943). This is in concurrence with the finding of an earlier study [34], where 37.6% of patients tested positive for at least one respiratory virus. Another study from Delhi, India, found that 35.2% of pediatric patients’ samples tested positive for respiratory viruses [35]. This is the first study from Eastern Uttar Pradesh, India, to detect multiple respiratory viruses in children aged below 5 years.

## 5. Conclusions and Future Perspectives

Respiratory viruses are the leading cause of hospitalization for acute lower respiratory tract infections in children younger than 5 years worldwide. In addressing the prevention and control of different respiratory viruses in India and globally, it is crucial to elucidate the regional prevalence, clinical profile, and seasonality of different viruses. Eastern UP shares an international border with Nepal and a national border with Bihar, due to which there are fair chances of cross-border transmission of respiratory infections. In addition, the climate is primarily humid subtropical, and may play a key role in differential transmission dynamics of respiratory viruses in the region. In our study, we estimated the prevalence and differential transmission dynamics, including seasonal patterns, of different respiratory viruses and their clinical presentations. We also reported the systematic distinct seasonal epidemic patterns of different respiratory viruses among children under five years of age. These seasonal patterns may be helpful for averting the disease burden of different respiratory viruses in the ARI/SARI pediatric population by the development of vaccines and/or therapies.

## 6. Limitations

In the present study, bacterial etiologies were not tested.

## Figures and Tables

**Table 1:** Socio-demographic and clinical characteristics of study population.

Patient’s Characteristics	ARI (n = 611)	SARI (n = 332)
Age (in months)	19.3 ± 16.5	9.5 ± 11.3
Male	406/611 (66.5)	214/332 (64.5)
Female	205/611 (33.5)	118/332 (35.5)
Weight (kg)	8.8 ± 3.4 (n = 308)	5.8 ± 2.9 (n = 284)
Similar illness in Family/Neighbourhood	21/611 (3.4)	11/332 (3.3)
Exposure to farm animals	39/611 (12.5)	17/332 (5.1)
Exposure to dead poultry birds	5/611 (0.81)	1/332 (0.3)
Smoker in Family	14/611 (2.29)	15/332 (4.51)
No. of family members sleeping in same room	2.14 ± 0.64	2.3 ± 0.64
Travel history prior 14 days to onset of symptom	1/611 (0.16)	0/332 (0)
History of fever in last 7 days from sample collection	603/611 (98.7)	325/332 (97.9)
History of cough in last 7 days from sample collection	609/611 (99.7)	323/332 (97.3)
Nasal Discharge	329/611 (53.9)	95/332 (28.6)
Chills/Rigors	33/611 (5.4)	0/332 (0)
History of Breathlessness	140/611 (22.9)	316/332 (95.2)
History of Vomiting	22/611 (53.9)	17/332 (28.6)
Abdominal Pain	5/611 (0.82)	8/332 (2.41)
Presented with a prior history of respiratory infection	41/611 (6.7)	263/332 (79.2)
Seizures	0/611 (0)	9/332 (2.71)
Diarrhoea	5/611 (0.82)	8/332 (2.41)
Wheezing	1/611 (0.16)	306/332 (92.2)
Crepitation	0/611 (0)	22/332 (6.6)
Nasal flaring	362/611 (59.3)	55/332 (16.6)
Apnea/sleep disorder	32/611 (5.2)	140/332 (42.2)
Decreased feeding	1/611 (0.16)	34/332 (10.2)
Antibiotics	509/611 (83.3)	332/332 (100)

**Table 2:** Positivity for different viral pathogens.

Viral Pathogens	ARI; n = 611 (%)	SARI; n = 332 (%)	Overall; n = 943 (%)
PIV	84 (13.75)	21 (6.33)	105 (11.13)
HAdV	56 (9.17)	26 (7.83)	82 (8.7)
RSV-B	26 (4.26)	42 (12.65)	68 (7.21)
Flu-A	36 (5.89)	10 (3.01)	46 (4.88)
SARS-CoV-2	23 (3.76)	5 (1.51)	28 (2.97)
hMPV	12 (1.96)	1 (0.30)	13 (1.38)
HRV	4 (0.42)	6 (0.64)	10 (1.06)
RSV-A	2 (0.33)	2 (0.60)	4 (0.42)
Flu-B	1 (0.16)	0 (0)	1 (0.11)
Total no. of virus positive samples	244 (40)	113 (34)	357 (37.9)

**Table 3:** Association between co-infection and clinical symptoms in ARI patients.

Clinical Symptoms	Co-Infection (+) = 34	Single-Infection (+) = 175	O.R. (95%C.I.)	*p*-Value
Nasal discharge	18 (52.9)	86 (49.1)	1.2 (0.6–2.4)	0.68
Chills/rigors	3 (8.8)	12 (6.9)	1.3 (0.4–4.9)	0.69
History of breathlessness in last 7 days	11 (32.4)	38 (21.7)	1.7 (0.8–3.9)	0.18
Sore throat	34 (100)	172 (98.3)	2.1 (0.4–11.4)	0.38
Body ache	2 (5.9)	5 (2.9)	2.1 (0.4–11.4)	0.38
History of vomiting in last 7 days	0 (0)	3 (1.7)	-	-
Abdominal pain	0 (0)	1 (0.6)	-	-
Presented with a prior history of respiratory infection	0 (0)	10 (5.7)	-	-
Nasal flaring	19 (55.9)	100 (57.1)	0.9 (0.4–2.0)	0.89
Apnea/sleep disorder	3 (8.8)	15 (8.6)	1.0 (0.3–3.8)	0.96

**Table 4:** Association between co-infection and clinical symptoms in SARI patients.

Clinical Symptoms	Co-Infection (+) = 12	Single-Infection (+) = 87	O.R. (95% C.I.)	*p*-Value
Nasal discharge	1 (8.3)	18 (20.7)	0.3 (0.0–2.9)	0.33
History of breathlessness in last 7 days	11 (91.7)	85 (97.7)	0.3 (0.0–3.1)	0.29
Sore throat	10 (83.3)	67 (77.0)	1.5 (0.3–7.4)	0.62
Body ache	1 (8.3)	5 (5.7)	1.5 (0.2–14.0)	0.73
Presented with a prior history of respiratory infection	10 (83.3)	71 (81.6)	1.1 (0.2–5.6)	0.89
Seizures	2 (16.7)	1 (1.1)	17.2 (1.4–207.1)	0.02
Wheezing	10 (83.3)	78 (89.7)	0.6 (0.1–3.1)	0.52
Nasal flaring	3 (25.0)	11 (12.6)	2.3 (0.5–9.8)	0.26
Apnea/sleep disorder	6 (50.0)	33 (37.9)	1.6 (0.5–5.5)	0.42
Decreased feeding	2 (16.7)	9 (10.3)	1.7 (0.3–9.2)	0.52
Mechanical ventilation	2 (16.7)	15 (17.2)	0.9 (0.2–4.8)	0.96
CPAP	8 (66.7)	32 (36.8)	3.4 (0.9–12.3)	0.05
Bronchiodilators	2 (16.7)	4 (4.6)	4.2 (0.7–25.6)	0.13
Abnormal * Pulse Rate	4 (36.4)	16 (20.5)	2.2 (0.6–8.5)	0.25
Axillary Temperature (>99.1 °F)	5 (45.5)	53 (69.7)	0.4 (0.1–1.3)	0.12
Oxygen Saturation (<90%)	3 (27.3)	34 (45.3)	0.5 (0.1–1.8)	0.27
Abnormal * Hemoglobin	8 (66.7)	48 (62.3)	1.2 (0.3–4.4)	0.77
Abnormal * WBC	6 (50.0)	31 (40.3)	1.5 (0.4–5.0)	0.52
Abnormal * Platelet	3 (25.0)	37 (48.1)	0.4 (0.1–1.4)	0.15
Abnormal * SGOT	2 (22.2)	14 (20.0)	1.1 (0.2–6.1)	0.88
Abnormal * SGPT	2 (22.2)	19 (27.5)	0.8 (0.1–4.0)	0.74
Abnormal * Serum Creatinine	3 (27.3)	35 (51.5)	0.4 (0.1–1.4)	0.15
Abnormal * BUN	2 (22.2)	14 (23.3)	0.9 (0.2–5.0)	0.94
Abnormal * Serum Sodium	2 (18.2)	17 (24.3)	0.7 (0.1–3.5)	0.66
Final Outcome Mortality	4 (36.4)	15 (19.2)	2.4 (0.6–9.3)	0.20
Hospitalization Days (Mean ± SD)	6.9 ± 3.6	11.4 ± 10.8	0.9 (0.8–1.0)	0.16

* Age specific.

## Data Availability

The data presented in this study are available on request from the corresponding author due to ethical reasons.

## Supplementary Materials

The following supporting information can be downloaded at: https://www.mdpi.com/article/10.3390/v17010027/s1,. Legends of supplementary Tables: Table S1: Forward and reverse Primers and TaqMan Probes; Table S2: Association between Viral Etiology (VE) and patient’s characteristics in ARI patients; Table S3: Association between Viral Etiology (VE) and patient’s characteristics in SARI patients; Table S4: Association between viral pathogens and clinical profile in ARI patients; Table S5: Association between viral pathogens and clinical profile in SARI patients. References [36,37,38,39,40,41,42,43] are cited in the Supplementary Materials.
