# Supplementary material for: Hospital-Based Surveillance of Respiratory Viruses Among Children Under Five Years of Age with ARI and SARI in Eastern UP, India
Source: Viruses. 2024 Dec 28;17(1):27. doi: 10.3390/v17010027 (PMC11769465; doi:10.3390/v17010027)
Supplement: Supplementary file 1 [file viruses-17-00027-s001.zip › viruses-3269513-supplementary.pdf]

Table S1: Forward and reverse Primers and TaqMan Probes

Primers and probes details	Sequences (5' to 3')	Working Conc. pmole/ul	Target (gene)	Reference
**InfA Forward**	GAC CRA TCC TGT CAC CTC TGA C	10	M gene	[36]
**InfA Reverse**	AGG GCA TTY TGG ACA AAK CGT CTA	10		
**InfA Probe**	**FAM-**TGC AGT CCT CGC TCA CTG GGC ACG-MGBNFQ	5		
**InfB Forward**	TCC TCA AYT CAC TCT TCG AGC G	10	NS gene	
**InfB Reverse**	CGG TGC TCT TGA CCA AAT TGG	10		
**InfB Probe1**	**NED-**CCA ATT CGA GCA GCT GAA ACT GCG GTG-MGBNFQ	5		
**Pdm H1 Forward**	GTG CTA TAA ACA CCA GCC TCC CATT	10	HA	
**Pdm H1 Reverse**	AGA **Y**GG GAC ATT CCT CAA TCC TG	10		
**Pdm H1 Probe**	**FAM-**ATA CAT CC**R** ATC ACA ATT GG**R** AAA TGT CCA AAMGBNFQ	5		
**A/H3 Forward**	AAG CAT TCC YAA TGA CAA ACC	10	HA	
**A/H3 Reverse**	ATT GCR CCR AAT ATG CCT CTA GT	10		
**A/H3 Probe**	**VIC-**CAG GAT CAC ATA TGG GSC CTG TCC CAG-MGBNFQ	5		
**RnaseP Forward**	AGA TTT GGA CCT GCG AGC G	10	RNase P	
**RnaseP Reverse**	GAG CGG CTG TCT CCA CAA GT	10		
**RnaseP Probe**	**VIC**-TTC TGA CCT GAA GGC TCT GCG CG-**MGBNFQ**	5		
**RSV A Forward**	AGA TCA ACT TCT GTC ATC CAG CAA	10	Nucleocapsid	[37]
**RSV A Reverse**	TTC TGC ACA TCA TAA TTA GGA G	10		
**RSV A Probe**	**FAM**-CAC CAT CCA ACG GAG CAC AGG AGA T-MGBNFQ	5		
**RSV B Forward**	AAG ATG CAA ATC ATA AAT TCA CAG GA	10	Nucleoprotein	
**RSV B Reverse**	TGA TAT CCA GCA TCT TTA AGT A	10		
**RSV B Probe**	**VIC-**TTT CCC TTC CTA ACC TGG ACA TA-MGBNFQ	5		
**Adeno-Forward**	GCC ACG GTG GGG TTT CTA AAC TT	10	Hexon	[38]
**Adeno-Reverse**	GCC CCA GTG GTC TTA CAT GCA CAT C	10		
**Adeno-Probe**	**VIC-**TGC ACC AGA CCC GGG CTC AGG TAC TCC GA-MGBNFQ	5		
**RhinoForward1**	GGT GTG AAG AGC CSC ATG TGC T	10	Polyprotein	[39]
**RhinoForward2**	GGT GTG AAG ACT CGC ATG TGC T	10		
**RhinoForward3**	GGG TGY GGA GAG YCT ANT GGC T	10		
**Rhino Reverse**	GGA CAC CCA AAG TAG TYG GTY C	10		

**Rhino Probe**	**NED-**CCG GCC CTG AAT GYG GCT AAY C- MGBNFQ	10		
**PIV1Forward**	ACC TAC AAG GCA ACA ACA TC	10	Haemaglutinin-Neuraminidase	[37]
**PIV1Reverse**	CTT CCT GCT GGT GTG TTA AT	10		
**PIV1Probe**	**FAM**–CAA ACG ATG GCT GAA AAA GGG A-MGBNFQ	5		
**PIV2Forward**	CCA TTT ACC TAA GTG ATG GAA	10	Haemaglutinin-Neuraminidase	
**PIV2Reverse**	CGT GGC ATA ATC TTC TTT TT	10		
**PIV2Probe**	**VIC**–AAT CGC AAA AGC TGT TCA GTC AC-MGBNFQ	5		
**PIV3Forward**	CCA GGG ATA TAY TAY AAA GGC AAA A	10	Haemaglutinin-Neuraminidase	
**PIV3Reverse**	CCG GGR CAC CCA GTT GTG	10		
**PIV3Probe**	**NED**-TGG RTG TTC AAG ACC TCC ATA YCC GAG AAA- MGBNFQ	5		
**PIV4Forward**	CAA AYG ATC CAC AGC AAA GAT TC	10	Haemaglutinin-Neuraminidase	[40]
**PIV4Reverse**	ATG TGG CCT GTA AGG AAA GCA	10		
**PIV4Probe**	**FAM**-GTA TCA TCA TCT GCC AAA TCG GCA ATT AAACA- MGBNFQ	5		
**hMPV A1A2 F**	CATATAAGCATGCTATATTAAAAGAGTCTC	10		[41]
**hMPV A1A2 R**	CCTATTTCTGCAGCATATTTGTAATCAG	10	Neucleocapsid	
**hMPVA1A2 Probe**	**FAM-**TGYAATGATGAGGGTGTCACTGCGGTTG	5		
**B HA BHA-188F**	AGA CCA GAG GGA AAC TAT GCC C	10	Haemaglutinin	[42]
**B HA BHA-270R**	TCC GGA TGT AAC AGG TCT GAC TT	10		
**Type B Victoria**	**VIC**-CAGACCAAAATGCACGGGGAAHATACC MGBNFQ	5		
**Type B Yamagata**	**FAM**-CAGRCCAATGTGTGTGGGGAYCACACC MGBNFQ	5		

References:

1. Laboratory Procedures for Detection of Influenza Virus in Specimens from Suspected Human Cases. National Centre for Disease Control (NCDC). Available online: https://ncdc.mohfw.gov.in/wp-content/uploads/2024/02/78851955051561442143.pdf (accessed on 7 October 2024).
2. Gunson, R.N.; Collins, T.; Carman, W. Real-time RT-PCR detection of 12 respiratory viral infections in four triplex reactions. *J. Clin. Virol.* **2005**, *33*, 341–344. https://doi.org/10.1016/j.jcv.2004.11.025.
3. Wong, S.; Pabbaraju, K.; Pang, X.L.; Lee, B.E.; Fox, J.D. Detection of a broad range of human adenoviruses in respiratory tract samples using a sensitive multiplex real-time PCR assay. *J. Med. Virol.* **2008**, *80*, 856–865. https://doi.org/10.1002/jmv.21136.
4. Brittain-Long, R.; Nord, S.; Olofsson, S.; Westin, J.; Anderson, L.M.; Lindh, M. Multiplex real-time PCR for detection of respiratory tract infections. *J. Clin. Virol.* **2008**, *41*, 53–56. https://doi.org/10.1016/j.jcv.2007.10.029.
5. Wang, C.; Arden, K.; Greer, R.; Sloots, T.; Mackay, I. A novel duplex real-time PCR for HPIV-4 detects co-circulation of both viral subtypes among ill children during 2008. *J. Clin. Virol.* **2012**, *54*, 83–85. https://doi.org/10.1016/j.jcv.2012.01.013.
6. Maertzdorf, J.; Wang, C.K.; Brown, J.B.; Quinto, J.D.; Chu, M.; de Graaf, M.; Hoogen, B.G.v.D.; Spaete, R.; Osterhaus, A.D.M.E.; Fouchier, R.A.M. Real-Time Reverse Transcriptase PCR Assay for Detection of Human Metapneumoviruses from All Known Genetic Lineages. *J. Clin. Microbiol.* **2004**, *42*, 981–986. https://doi.org/10.1128/jcm.42.3.981-986.2004.

7. WHO. Information Forthe Molecular Detectionof Influenza Viruses. World Health Organisation. Available online: https://cdn.who.int/media/docs/default-source/influenza/molecular-detention-of-influenza-viruses/protocols_influenza_virus_detection_feb_2021.pdf (accessed on 7 October 2024).

Table S2: Association between Viral Etiology (VE) and patient's characteristics in ARI patients

Patient's characteristics	VE (+)=209	VE (-)=402	O.R. (95% C.I.)	*P-value*	A.O.R. (95% C.I.)	*P-value*
**Age (months)**						
**1-12**	106 (50.7)	202 (50.2)	(Ref.)	-		
**13-24**	53 (25.3)	93 (23.1)	1.1 (0.7-1.6)	*0.69*	-	-
**25-60**	50 (24.0)	107 (26.7)	0.9(0.6-1.3)	*0.58*		
**Gender**						
**Male**	146 (69.9)	260 (64.7)	(Ref.)	-	-	-
**Female**	63 (30.1)	142 (35.3)	0.8 (0.6-1.1)	*0.20*		
**Similar illness in family or neighborhood**	12 (5.7)	9 (2.2)	2.7 (1.1-6.4)	*0.03*	1.4 (0.5-3.7)	*0.52*
**Exposure to farm animals**	26 (12.4)	13 (3.2)	4.3 (2.1-8.5)	*<0.01*	3.4 (1.6-7.1)	*<0.01*
**Exposure to dead poultry birds**	3 (1.4)	2 (0.5)	2.9 (0.5-17.6)	*0.24*	-	-
**Smoker in Family**	10 (4.8)	4 (1.0)	5.0 (1.5-16.1)	*<0.01*	2.5 (0.7-8.8)	*0.15*
**No. of family members sleeping in same room (Mean±SD)**	(2.1 ± 0.7)	(2.1 ± 0.6)	1.0 (0.7 - 1.3)	*0.97*	-	-
**Nasal Discharge**	104 (49.8)	225 (56.0)	1.6 (0.8-3.3)	*0.82*	-	-
**Chills/Rigors**	15 (7.2)	18 (4.5)	1.0(0.8-3.3)	*0.63*	-	-
**History of Breathlessness in last 7 days**	49 (23.4)	91 (22.6)	1.0 (0.7-1.6)	*0.82*	-	-
**Sore throat**	206 (98.6)	398 (99.0)	0.7 (0.2-3.1)	*0.63*	-	-
**Body ache**	7 (3.3)	19 (4.7)	0.7 (0.3-1.7)	*0.43*	-	-
**History of Vomiting in last 7 days**	3 (1.4)	19 (4.7)	0.3 (0.1-1.0)	*0.05*	-	-
**Abdominal Pain**	1 (0.5)	4 (1.0)	0.5(0.1-4.3)	*0.05*	-	-
**Presented with a prior history of respiratory infection**	10 (4.8)	31 (7.7)	0.6 (0.3-1.3)	*0.17*	-	-
**Nasal flaring**	119 (56.9)	243 (60.4)	0.9 (0.6-1.2)	*0.40*	-	-
**Apnea/sleep disorder**	18 (8.6)	14 (3.5)	2.6 (1.3-5.4)	*<0.01*	2.3 (1.1 - 4.9)	*0.02*
**Antibiotics**	167 (79.9)	342 (85.1)	0.7 (0.5-1.1)	*0.11*	-	-

Table S3: Association between Viral Etiology (VE) and patient's characteristics in SARI patients

Patient's characteristics	VE (+) = 99	VE (-) = 233	O.R. (95% C.I.)	*P-value*	A.O.R. (95% C.I.)	*P-value*
**Age (months)**						
**1-12**	85 (85.9)	171 (73.4)	1.0 (Ref.)			
**13-24**	10 (10.1)	39 (16.7)	0.5 (0.2-1.1)	*0.08*	-	-
**25-60**	4 (4.0)	23 (9.9)	0.3 (0.1-1.0)	*0.06*		
**Gender**						
**Male**	65 (65.7)	149 (63.9)	1.0 (Ref.)	-	-	-
**Female**	34 (34.3)	84 (36.1)	0.9 (0.6-1.5)	*0.77*		
**Similar illness in family or neighborhood**	5 (5.1)	6 (2.6)	2.0 (0.6-6.8)	*0.26*	-	-
**Exposure to farm animals**	12 (12.1)	5 (2.1)	6.3(2.2-18.4)	*<0.01*	4.3(1.3-14.0)	*0.02*
**Smoker in Family**	11 (11.1)	4 (1.7)	7.2 (2.2-23.1)	*<0.01*	9.2 (2.4-35.7)	*<0.01*
**No. of family members sleeping in same room (Mean±SD)**	2.3 ± 0.6	2.3 ± 0.7	1.1 (0.7-1.5)	*0.78*	-	-
**Nasal Discharge**	19 (19.2)	76 (32.6)	0.5 (0.3-0.9)	*0.01*	0.6 (0.3-1.2)	*0.13*
**History of Breathlessness in last 7 days**	96 (97.0)	220 (94.4)	1.9 (0.5-6.8)	*0.33*	-	-
**Sore throat**	77 (77.8)	212 (91.0)	0.3 (0.2-0.7)	*<0.01*	0.3 (0.1-0.7)	*<0.01*
**Body ache**	6 (6.0)	10 (4.3)	1.4 (0.5-4.1)	*0.49*	-	-
**History of Vomiting in last 7 days**	3 (3.0)	14 (6.0)	0.5 (0.1-1.7)	*0.27*	-	-
**Abdominal Pain**	3 (3.0)	5 (2.1)	1.4 (0.3-6.1)	*0.63*	-	-
**Presented with a prior history of respiratory infection**	81 (81.8)	182 (78.1)	1.3 (0.7-2.3)	*0.45*	-	-
**Seizures**	3 (3.0)	6 (2.6)	1.2 (0.3-4.8)	*0.82*	-	-
**Diarrhoea**	3 (3.0)	5 (2.1)	1.4 (0.3-6.1)	*0.63*	-	-
**Wheezing**	88 (88.9)	218 (93.6)	0.5 (0.2-1.2)	*0.15*	-	-
**Crepitation**	6 (6.0)	16 (6.9)	0.9 (0.3-2.3)	*0.79*	-	-
**Nasal flaring**	14 (14.1)	41 (17.6)	0.8 (0.4-1.5)	*0.44*	-	-
**Apnea/sleep disorder**	39 (39.4)	101 (43.7)	0.8 (0.5-1.4)	*0.51*	-	-
**Decreased Feeding**	11 (11.1)	23 (9.9)	1.1 (0.5-2.4)	*0.73*	-	-
**Mechanical Ventilation**	17 (17.2)	50 (21.5)	0.8 (0.4-1.4)	*0.37*	-	-
**Oxygen**	96 (97.0)	230 (98.7)	0.4 (0.1-2.1)	*0.29*	-	-
**CPAP**	40 (40.4)	71 (30.5)	1.5 (0.9-2.5)	*0.08*	-	-

**Bronchiodilators**	6 (6.0)	3 (1.3)	4.9 (1.2-20.2)	*0.02*	6.5 (0.6-65.8)	*0.11*
**Abnormal* Respiratory Rate/min**	26 (32.9)	51 (27.4)	1.3 (0.7-2.3)	*0.368*	-	-
**Abnormal* Pulse Rate**	20 (22.5)	43 (21.4)	1.1 (0.6-1.9)	*0.84*	-	-
**Abnormal* Axillary Temperature (>99.1°F)**	58 (66.7)	100 (51.5)	1.9 (1.1-3.2)	*0.02*	1.8 (1.0-3.1)	*0.05*
**Abnormal* Oxygen Saturation (<90%)**	37 (43.2)	100 (50.0)	0.8 (0.5-1.3)	*0.28*	-	-
**Abnormal* Hemoglobin**	56 (62.9)	148 (74.4)	0.6 (0.3-1.0)	*0.05*	-	-
**Abnormal* WBC**	37 (41.6)	82 (41.4)	1.0 (0.6-1.7)	*0.98*	-	-
**Abnormal* Platelet**	40 (44.1)	78 (39.6)	1.2 (0.8-2.1)	*0.40*	-	-
**Abnormal* CRP**	74 (98.7)	168 (98.8)	0.9 (0.1-9.9)	*0.92*	-	-
**Abnormal* SGOT**	16 (20.3)	45 (24.5)	0.8 (0.4-1.5)	*0.46*	-	-
**Abnormal* SGPT**	21 (26.9)	57 (31.0)	0.8 (0.5-1.5)	*0.51*	-	-
**Abnormal* Serum Albumin**	7 (9.9)	20 (12.0)	0.8 (0.3-2.0)	*0.63*	-	-
**Abnormal* Serum Alkaline Phosphatase**	46 (59.7)	97 (53.0)	1.3 (0.8-2.3)	*0.32*	-	-
**Abnormal* Serum Creatinine**	38 (48.1)	95 (52.5)	0.8 (0.5-1.4)	*0.52*	-	-
**Abnormal* BUN**	16 (23.2)	40 (24.2)	0.9 (0.5-1.8)	*0.86*	-	-
**Abnormal* Serum Sodium**	19 (23.5)	44 (25.4)	0.9 (0.5-1.6)	*0.73*	-	-
**Abnormal* Serum Potassium**	11 (13.6)	38 (22.0)	0.6 (0.3-1.2)	*0.12*	-	-

*Age specific

Reference values for common laboratory tests (https://www.accp.com/docs/sap/Lab_Values_Table_PedSAP.pdf) [Accessed on 19 September 2024)

Respiratory rate and Pulse rate (https://www.health.ny.gov/professionals/ems/pdf/assmttools.pdf) [Accessed on 19 September 2024)

Axillary Temperature [43]

1. Leduc, D.; Woods, S. Community Paediatrics Committee Temperature measurement in paediatrics. *Paediatr. Child Health* **2000**, *5*, 273–276. https://doi.org/10.1093/pch/5.5.273.

Table S4: - Association between viral pathogens and clinical profile in ARI patients

Clinical parameters		SARS COV 2 (n=23)	INF A (n=36)	INF B (n=1)	HMPV (n=12)	RSV A (n=2)	RSV B (n=26)	PIV 1 (n=65)	PIV 2 (n=14)	PIV 4 (n=5)	ADENOVIRUS (n=56)	HUMAN RHINO VIRUS (n=4)
Fever	Present	23 (100)	23 (63.9)	1 (100)	12 (100)	2 (100)	26 (100)	65 (100)	14 (100)	5 (100)	56 (100)	4 (100)
Cough	Present	23 (100)	23 (63.9)	1 (100)	12 (100)	2 (100)	26 (100)	64 (98.5)	14 (100)	5 (100)	56 (100)	4 (100)
Nasal Discharge	Present	11 (47.8)	18 (50)	1 (100)	9 (75)	1 (50)	15 (57.7)	33 (50.8)	6 (42.9)	2 (40)	26 (46.4)	1 (25)
Chills/Rigors	Present	1 (4.3)	5 (13.9)	0 (0)	1 (8.3)	1 (50)	4 (15.4)	2 (3.1)	0 (0)	1 (20)	3 (5.4)	1 (25)
Sore Throat	Present	23 (100)	36 (100)	1 (100)	11 (91.7)	2 (100)	25 (96.2)	64 (98.5)	14 (100)	5 (100)	56 (100)	4 (100)
Breathlessness	Present	3 (13.0)	3 (8.3)	1 (100)	2 (16.7)	0 (0)	7 (26.9)	19 (29.2)	4 (28.6)	1 (20)	19 (33.9)	1 (25)
Body ache	Present	1 (4.3)	0 (0)	0 (0)	0 (0)	0 (0)	1 (3.8)	3 (4.6)	1 (7.1)	0 (0)	3 (53.4)	0 (0)
Vomiting	Present	0 (0)	0 (0)	0 (0)	1 (8.3)	0 (0)	1 (3.8)	0 (0)	0 (0)	0 (0)	1 (1.8)	0 (0)
Abdominal Pain	Present	0 (0)	0 (0)	0 (0)	0 (0)	0 (0)	0 (0)	0 (0)	0 (0)	0 (0)	1 (1.8)	0 (0)
Past History of Respiratory Infection/ similar complain	Present	0 (0)	3 (8.3)	0 (0)	0 (0)	0 (0)	1 (3.8)	0 (0)	0 (0)	0 (0)	5 (8.9)	0 (0)
Seizures	Present	0 (0)	0 (0)	0 (0)	0 (0)	0 (0)	0 (0)	0 (0)	0 (0)	0 (0)	0 (0)	0 (0)
Diarrhoea	Present	0 (0)	0 (0)	0 (0)	0 (0)	0 (0)	0 (0)	0 (0)	0 (0)	0 (0)	1 (1.8)	0 (0)
Wheeze	Present	0 (0)	0 (0)	0 (0)	0 (0)	0 (0)	0 (0)	0 (0)	0 (0)	0 (0)	0 (0)	0 (0)
Crepitation	Present	0 (0)	0 (0)	0 (0)	0 (0)	0 (0)	0 (0)	0 (0)	0 (0)	0 (0)	0 (0)	0 (0)
Nasal Flaring	Present	16 (69.6)	26 (72.2)	0 (0)	6 (50)	1 (50)	13 (50)	32 (49.2)	8 (57.1)	3 (60)	31 (55.4)	3 (75)
Apnoea	Present	1 (4.3)	0 (0)	0 (0)	2 (16.7)	0 (0)	2 (7.7)	5 (7.7)	2 (14.3)	1 (20)	6 (10.7)	2 (50)
Decreased Feeding	Present	16 (69.6)	0 (0)	0 (0)	0 (0)	0 (0)	0 (0)	1 (1.5)	0 (0)	0 (0)	0 (0)	0 (0)
Antibiotics	Present	1 (4.3)	30 (83.3)	1 (100)	9 (75)	2 (100)	23 (88.5)	48 (73.8)	14 (100)	5 (100)	42 (75)	3 (75)

Table S5- Association between viral pathogens and clinical profile in SARI patients

Clinical parameters		SARSCOV2 (n=5)	INF A (n=10)	HMPV (n=1)	RSV A (n=2)	RSV B (n=42)	PIV 1 (n=14)	PIV 2 (n=4)	PIV 4 (n=3)	ADENOVIRUS (n=26)	HUMAN RHINOVIRUS (n=6)
Fever	Present	5 (100)	10 (100)	1 (100)	2 (100)	40 (95.2)	12 (85.7)	3 (75)	3 (100)	26 (100)	6 (100)
Cough	Present	5 (100)	9 (90)	1 (100)	2 (100)	40 (95.2)	12 (85.7)	4 (100)	3 (100)	25 (96.2)	6 (100)
Nasal Discharge	Present	0 (0)	1 (10)	0 (0)	0 (0)	10 (23.8)	1 (7.1)	1 (25)	0 (0)	6 (23.1)	1 (16.7)
Chills/Rigors	Present	0 (0)	0 (0)	0 (0)	0 (0)	0 (0)	0 (0)	0 (0)	0 (0)	0 (0)	0 (0)
Sore Throat	Present	4 (80)	8 (80)	0 (0)	0 (0)	33 (78.6)	9 (64.3)	3 (75)	2 (66.7)	23 (88.5)	6 (100)
Breathlessness	Present	5 (100)	9 (90)	1 (100)	2 (100)	41 (97.6)	14 (100)	3 (75)	3 (100)	25 (96.2)	6 (100)
Body ache	Present	0 (0)	1 (10)	0 (0)	0 (0)	3 (7.1)	0 (0)	1 (25)	0 (0)	2 (7.7)	0 (0)
Vomiting	Present	0 (0)	0 (0)	0 (0)	0 (0)	2 (4.8)	0 (0)	0 (0)	0 (0)	1 (3.8)	0 (0)
Abdominal Pain	Present	0 (0)	0 (0)	1 (100)	0 (0)	0 (0)	2 (14.3)	0 (0)	0 (0)	0 (0)	0 (0)
Past history of Respiratory Infection/ similar complain	Present	4 (80)	8 (80)	1 (100)	1 (50)	38 (90.5)	7 (50)	2 (50)	3 (100)	23 (88.5)	6 (100)
Seizures	Present	0 (0)	3 (30)	0 (0)	0 (0)	0 (0)	0 (0)	1 (25)	0 (0)	1 (3.8)	0 (0)
Diarrhoea	Present	0 (0)	0 (0)	1 (100)	0 (0)	0 (0)	2 (14.3)	0 (0)	0 (0)	0 (0)	0 (0)
Wheeze	Present	5 (100)	10 (100)	1 (100)	2 (100)	35 (83.3)	14 (100)	4 (100)	3 (100)	22 (84.6)	4 (66.7)
Crepitation	Present	0 (0)	0 (0)	0 (0)	0	2 (4.8)	0 (0)	0 (0)	1 (33.3)	2 (7.7)	1 (16.7)
Nasal Flaring	Present	1 (20)	2 (20)	0 (0)	1 (50)	2 (4.8)	5 (35.7)	1 (25)	0 (0)	4 (15.4)	1 (16.7)
Apnea	Present	1 (20)	5 (50)	0 (0)	1 (50)	19 (45.2)	2 (14.3)	2 (50)	1 (33.3)	10 (38.5)	4 (66.7)

Decreased Feeding	Present	0 (0)	0 (0)	0 (0)	0 (0)	5 (11.9)	1 (7.1)	1 (25)	0 (0)	4 (15.4)	1 (16.7)
Mechanical Ventilation	Present	0 (0)	0 (0)	0 (0)	0 (0)	4 (9.5)	2 (14.3)	1 (25)	1 (33.3)	9 (34.6)	2 (33.3)
Oxygen	Present	5 (100)	10 (100)	0 (0)	2 (100)	40 (95.2)	14 (100)	4 (100)	3 (100)	25 (96.2)	6 (100)
CPAP	Present	4 (80)	3 (30)	0 (0)	0 (0)	14 (33.3)	1 (7.1)	1 (25)	2 (66.7)	11 (42.3)	3 (50)
Steroid	Present	0 (0)	1 (10)	0 (0)	0 (0)	0 (0)	1 (7.1)	0 (0)	1 (33.3)	1 (3.8)	1 (16.7)
Bronchiodilators	Present	0 (0)	1 (10)	0 (0)	0 (0)	1 (2.4)	2 (14.3)	2 (50)	0 (0)	2 (7.7)	0 (0)
Chest X-Ray	Present	3 (60)	9 (90)	1 (100)	2 (100)	38 (90.5)	5 (35.7)	2 (50)	3 (100)	23 (88.5)	6 (100)
Heterogeneous capacities	Present	3 (60)	8 (80)	1 (100)	1 (50)	32 (76.2)	4 (28.6)	2 (50)	2 (66.7)	17 (65.4)	5 (83.3)
Admitted in ICU	Present	5 (100)	10 (100)	1 (100)	2 (100)	42 (100)	14 (100)	4 (100)	3 (100)	26 (100)	6 (100)
Sepsis	Present	2 (40)	0 (0)	0 (0)	0 (0)	13 (31.0)	4 (28.6)	0 (0)	1 (33.3)	7 (26.9)	1 (16.7)
Outcome	Discharge	4 (80)	6 (60)	1 (100)	2 (100)	33 (78.6)	11 (78.6)	4 (100)	2 (66.7)	13 (50.0)	0 (0)
	Expire	1 (20)	1 (10)	0 (0)	0 (0)	5 (11.9)	2 (14.3)	0 (0)	1 (33.3)	10 (38.5)	4 (66.7)
	LAMA	0 (0)	3 (30)	0 (0)	0 (0)	4 (9.5)	1 (7.1)	0 (0)	0 (0)	3 (11.5)	2 (33.3)
Respiratory Rate		51.2±13.9	51.9±10.9	#	#	53.2±13.5	45.4±15.2	40.6±12.0	54.1±13.4	50.4±14.1	44.7±6.4
Pulse Rate		143.1±20.5	143.7±20.4	#	#	144.5±20.2	141.3±18.9	138.0±18.5	143.9±20.3	143.2±20.2	143.8±13.4
Oxygen Saturation		94.4±5.4	94.3±5.6	#	#	94.3±5.3	93.9±2.7	94.0±3.0	94.3±5.5	94.2±5.4	94.8±1.0
Haemoglobin		9.9±1.7	9.9±1.7	#	#	10.0±1.8	9.6±1.7	9.9±1.6	10.0±1.7	9.9±1.7	9.5±1.4
WBC		20033.2±33936.9	18770.6±23814.9	#	#	19177.0±24799.7	18852.1±30262.2	29890.8±71970.5	19186.0±25740.4	20423.8±36689.1	14740.0±6377.5
Neutrophil		52.3±19.4	51.5±19.3	#	#	51.9±19.5	50.2±21.6	49.846±21.73	51.7±19.4	51.4±19.8	55.9±20.5
Lymphocytes		40.0±47.9	40.6±49.5	#	#	40.2±53.6	352.0±2288.8	589.2±3042.9	36.7±16.5	112.7±1104.4	37.0±19.5
Mid Value		8.3±5.1	8.3±5.3	#	#	8.5±5.5	7.5±4.7	8.5±3.8	8.7±5.5	8.4±5.2	6.6±1.6

Eosinophil		1.5±1.5	1.5±1.6	#	#	1.6±1.6	2.0±5.3	2.5±6.9	1.6±1.6	1.6±2.9	1.0±0.5
Platelet		382459.1±361986.6	381125.1±370180.3	#	#	388041.4±397709.8	300954.8±167893.1	314651.2±164556.1	397198.2±410394.8	372554.0±355786.7	255200.0±140116.7
ESR		27.7±11.6	27.7±11.4	#	#	27.7±11.7	25.9±10.6	20.8±10.3	27.5±11.6	27±11.5	24.0±9.4
CRP		41.5±69.8	42.3±70.8	#	#	44.8±75.6	19.9±29.7	19.5±28.8	47.4±77.8	39.9±68.6	26.8±19.1
Serum Bilirubin Total		0.6±0.8	0.6±0.9	#	#	0.6±0.9	0.4±0.3	0.5±0.3	0.7±1.0	0.6±0.8	0.3±0.1
Serum Bilirubin Direct		0.5±2.2	0.5±2.3	#	#	0.5±2.5	0.6±2.4	0.3±0.2	0.4±2.2	0.5±2.2	0.2±0.04
Serum Bilirubin Indirect		0.4±1.1	0.4±1.2	#	#	0.5±1.3	0.2±0.2	0.2±0.2	0.5±1.3	0.4±1.1	0.2±0.1
SGOT		78.8±105.0	79.7±109.1	#	#	82.5±117.3	59.4±53.4	58.4±3767.4	84.0±120.4	77.4±106.6	116.8±82.9
SGPT		57.7±89.8	57.1±90.2	#	#	58.8±96.8	44.2±47.1	51.4±58.8	60.2±99.7	56.0±88.2	56.1±29.1
Serum Proteins		5.9±2.5	5.8±2.6	#	#	5.8±2.8	5.7±1.8	N/A	5.9±2.8	5.9±2.6	6.1±0.7
Serum Albumin		4.0±0.9	4.0±0.9	#	#	4.0±0.9	3.8±1.1	N/A	4.0±0.8	4.0±0.9	4.6±0.2
Serum Globulin		1.8±0.9	1.8±0.9	#	#	1.8±0.9	2.1±1.1	N/A	1.8±0.9	1.8±0.9	1.6±0.4
Serum Alkaline Phosphatase		442±233.2	429.6±220.6	#	#	414.5±212.5	484.5±484.1	560.2±595.4	417.2±207.9	443.5±301.8	354.6±163.1
Serum Urea		34.1±24.6	34.2±25.4	#	#	34.6±26.7	33.9±22.2	39.6±25	35.1±26.9	35±25.9	32.8±14.2
Serum Creatinine		0.8±0.6	0.8±0.6	#	#	0.8±0.6	0.7±0.6	0.8±0.7	0.8±0.6	0.8±0.6	0.8±0.2
BUN		15.4±7.7	15.5±7.9	#	#	15.5±7.9	19.3±25.6	0.8±0.7	15.7±7.7	16.2±12.3	15.3±6.5
Serum Sodium		145.0±87.0	145.1±90.9	#	#	146.2±100.2	153.9±169.7	138.7±8.0	140.2±13.1	144.3±87.5	139.8±4.2

Serum Potassium		4.7±1.0	4.8±1.0	#	#	4.8±1.0	4.5±1.4	4.7±1.0	4.9±0.9	4.7±1.0	4.4±0.3
Serum Calcium		1.9±8.3	2.0±8.7	#	#	2.1±9.6	1.5±1.2	1.4±0.3	2.1±9.9	1.9±8.4	1.1±0.2
